# Role of Orbitofrontal Cortex and Differential Effects of Acute and Chronic Stress on Motor Impulsivity Measured With 1-Choice Serial Reaction Time Test in Male Rats

**DOI:** 10.1093/ijnp/pyac062

**Published:** 2022-09-10

**Authors:** Milena Girotti, Flavia R Carreno, David A Morilak

**Affiliations:** Department of Pharmacology and Center for Biomedical Neuroscience, University of Texas Health Science Center at San Antonio, San Antonio, TX, USA; Department of Pharmacology and Center for Biomedical Neuroscience, University of Texas Health Science Center at San Antonio, San Antonio, TX, USA; Department of Pharmacology and Center for Biomedical Neuroscience, University of Texas Health Science Center at San Antonio, San Antonio, TX, USA; South Texas Veterans Health Care System, San Antonio, TX, USA

**Keywords:** OFC, stress, 1-CSRTT, motor impulsivity

## Abstract

**Background:**

Deficits in motor impulsivity, that is, the inability to inhibit a prepotent response, are frequently observed in psychiatric conditions. Several studies suggest that stress often correlates with higher impulsivity. Among the brain areas affected by stress, the orbitofrontal cortex (OFC) is notable because of its role in impulse control. OFC subregions with unique afferent and efferent circuitry play distinct roles in impulse control, yet it is not clear what OFC subregions are engaged during motor impulsivity tasks.

**Methods:**

In this study we used a rodent test of motor impulsivity, the 1-choice serial reaction time test, to explore activation of OFC subregions either during a well-learned motor impulsivity task or in a challenge task with a longer wait time that increases premature responding. We also examined the effects of acute inescapable stress, chronic intermittent cold stress and chronic unpredictable stress on motor impulsivity.

**Results:**

Fos expression increased in the lateral OFC and agranular insular cortex during performance in both the mastered and challenge conditions. In the ventral OFC, Fos expression increased only during challenge, and within the medial OFC, Fos was not induced in either condition. Inescapable stress produced a transient effect on premature responses in the mastered task, whereas chronic intermittent cold stress and chronic unpredictable stress altered premature responses in both conditions in ways specific to each stressor.

**Conclusions:**

These results suggest that different OFC subregions have different roles in motor impulse control, and the effects of stress vary depending on the nature and duration of the stressor.

**Significance Statement**

Inability to refrain from acting impulsively can have serious consequences in everyday life. Impulsivity is a component of many psychiatric conditions, and symptoms related to poor decision-making and increased impulsivity can be aggravated by stress. The specific neurobiological systems responsible for impulse control remain to be understood, as does the impact that different types of stressors may have on these systems. Here we provide evidence that specific subregions of the orbitofrontal cortex (OFC) are engaged during inhibition of impulsive action and that chronic stress can directly interfere with the control of impulsive responding. Future work will probe the neuronal circuits underlying the inhibition of impulsive action in OFC and how they are affected by stress, informing potential interventions for deficits of impulse control.

## Introduction

Impulsive responding, defined as a deficit in behavioral inhibition characterized by an inability to restrain a response despite potentially negative consequences, is prevalent in several psychiatric conditions, notably, attention deficit hyperactivity disorder, obsessive compulsive disorder, and addiction-related behaviors. Impulsivity is also associated with stress-related disorders, such as posttraumatic stress disorder, raising the possibility that stress may induce or exacerbate impulsive symptoms (Chamberlain et al., 2006, 2007; Morein-Zamir et al., 2010; Potenza and de Wit, 2010; Walker et al., 2018). Trait impulsivity has been shown to interact with stress to precipitate maladaptive behaviors such as substance abuse, gambling, and compulsions (Oswald et al., 2007; Sinha, 2008; Grubbs and Chapman, 2019; Raio et al., 2020; Albertella et al., 2021; Verdejo-Garcia and Albein-Urios, 2021). Chronic stress has also been shown in rodents to increase addictive behaviors and self-administration of drugs of abuse (Sinha, 2001; Lu et al., 2003; Ansell et al., 2012; Hamilton et al., 2014). Despite this evidence from both human and animal models, analysis of the effects of qualitatively distinct stressors on impulsivity is lacking.

Impulsive responding is not a unitary concept but encompasses at least 2 behavioral constructs, “impulsive action” or motor impulsivity, the inability to inhibit a prepotent response, and “impulsive choice,” selection of a small, immediate reward in favor of a larger, delayed reward, that are controlled by different neural mechanisms (Evenden, 1999; Winstanley et al., 2006). Among different tests of impulsive responding with a focus on motor inhibition, the 4- and 5-choice serial reaction time tests are used in humans and rodents, respectively, to measure the ability to suppress responses in anticipation of a reward (wait impulsivity) (Bari et al., 2008; Worbe et al., 2014). In the 5-choice serial reaction time test (CSRTT), rats are trained to detect brief flashes of light presented randomly in 1 of 5 holes and make a nose-poke response in the correct location to receive a food reward. Premature responses made before presentation of the stimulus are regarded as motor impulsivity (Eagle and Baunez, 2010; Sosa et al., 2021). However, because the stimulus can appear in any of 5 apertures, the animal is additionally tasked with an attentional burden that may confound effects on impulsivity. Thus, a simplified version of this task was developed, the 1-CSRTT, where only 1 stimulus hole is available, reducing attentional demand to focus more directly on impulsivity (Dalley et al., 2002; Anastasio et al., 2011).

The OFC plays a critical role in behavioral inhibitory control and decision-making, because damage in this area often leads to reduced sensitivity to negative outcomes, dysregulated affect, and decreased impulse control (Berlin et al., 2004; Szatkowska et al., 2007; Solbakk et al., 2014). A role of OFC in impulsive action has also been documented in rodents by lesion studies (Chudasama et al., 2003) and more recently by recording neuronal activity during a stop-signal task in macaques (Balasubramani et al., 2020). One limitation of lesions is that they are often extensive and not confined to specific subregions of the OFC. Yet, it is becoming increasingly evident that different subregions of the OFC have distinct roles in flexibility, decision-making, and impulse control (Mar et al., 2011; Izquierdo, 2017; Hervig et al., 2020). For example, differences in functional connectivity of medial OFC and lateral OFC circuits make distinct contributions to pathophysiology in major depression and obsessive compulsive disorder (for review, see (Fettes et al., 2017)). However, much is still unknown with respect to specific OFC areas involved in motor impulsivity. In this study, we used a modified version of the 1-CSRTT to explore activation of different subregions of the OFC during motor inhibition tasks with different inhibitory demands: during performance on a well-learned task and during a novel challenge task with a longer wait time. We then examined the effects of 3 qualitatively different stressors—severe acute stress, chronic metabolic stress, and chronic psychogenic stress—on motor impulsivity in rats.

## Methods

### Animals

A total of 62 adult male Sprague-Dawley rats (Envigo, 225–249 g on arrival) were housed 2 per cage on a 12/12-hour light cycle (lights on at 7:00 am) with food and water ad libitum except during the period of food restriction. Animals acclimated for 1 week before experimental procedures began. Experiments took place between 9:00 am and 2:00 pm. All procedures were in accordance with National Institutes of Health guidelines and approved by the University of Texas Health San Antonio (UTHSA) Institutional Animal Care and Use Committee. These experiments represent early-stage work to develop and optimize the 1-CSRTT behavioral test and were conducted in male rats only to replicate procedures in the studies that provided the model for our protocol, which used only male rats. For future work, we are establishing protocols to measure motor impulsivity and sensitivity to stress in female rats. Group sizes were determined a priori by power analysis using G*Power, with α= .05 and power = 0.8.

### 1-Choice Serial Reaction Time Task

**Figure 1.**
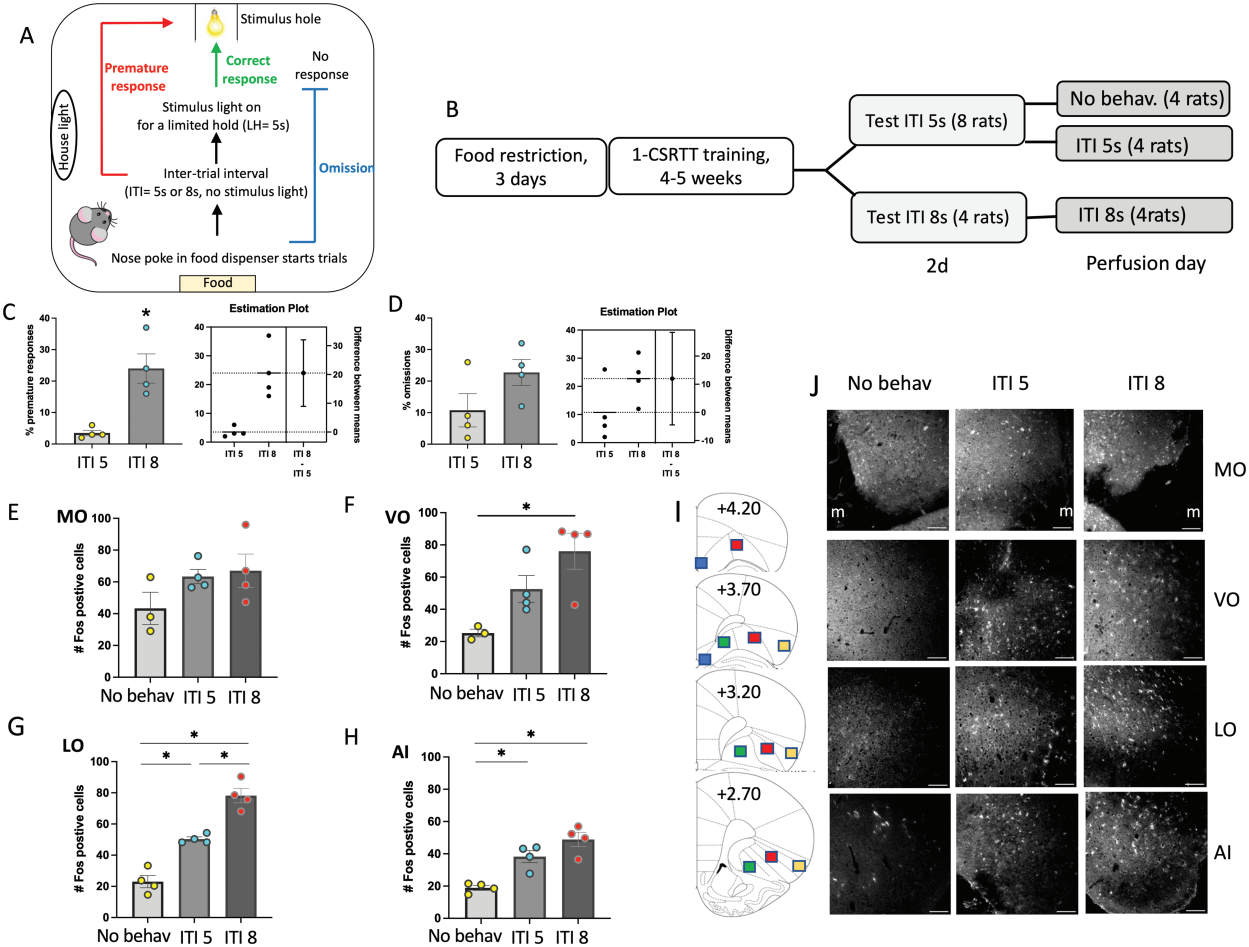
Performance in the 1-choice serial reaction time test (1-CSRTT) under maintenance (intertrial interval (ITI) 5 sec) or challenge conditions (ITI 8 sec) increases Fos expression in the orbitofrontal cortex (OFC). (A) Diagrammatic depiction of the 1-CSRTT. (B) Experimental timeline. (C–D) Premature responses and omissions in the 1-CSRTT, tested 1 hour before perfusion for Fos immunohistochemistry. (E–H) Fos-positive cells counted in the medial OFC (MO), ventral OFC (VO), lateral OFC (LO), and agranular insular cortex (AI) in cage control rats (no behavior) or after performance in the 1-CSRTT under well-trained conditions (ITI 5) or at a novel and longer ITI (ITI 8). (I) Diagrammatic representation of regions analyzed in the MO (blue squares), VO (green squares), LO (red squares), and AI (yellow squares). (J) Representative images of Fos immunofluorescence in the OFC after no behavior and after performance in the ITI 5 and ITI 8 conditions. Scale bar = 100 µm; m = midline. *P < .05, n = 3–4 per group.

To measure motor impulsivity, we employed the 1-CSRTT (Fig. 1A) (Dalley et al., 2002; Anastasio et al., 2011). In the original version of this test, rats were trained in a 5-CSRTT chamber, but the visual stimulus was exclusively presented in 1 aperture. However, the animals may still be distracted by and make “incorrect” responses in the unlit holes, and these responses must be considered in the evaluation of results. Thus, we modified the test by utilizing a chamber with only 1 stimulus hole, thereby eliminating “incorrect choices,” further reducing attentional demand, and focusing the test more directly on motor inhibition.

#### Apparatus

The 1-CSRTT was conducted in a single-hole nose-poke operant chamber (12 inches wide × 10 inches deep × 12 inches high) with a non-shock grid floor, enclosed within a sound attenuating cabinet (23 inches wide × 24 inches high × 20 inches deep) (Coulbourn Instruments Whitehall, PA, USA). The operant chamber was equipped with a house light and a food magazine that delivered 45 mg dustless sugar pellets (Bioserv) positioned on the wall opposite the stimulus hole (Fig. 1A). Both the stimulus hole and food magazine were equipped with a sensor to detect nose poke entries. The chambers were controlled by a Habitest Linc Tower with Graphic State 4 software (Coulbourn Instruments).

#### Training and Testing

The protocol for habituation and training were essentially as described in (Dalley et al., 2002; Anastasio et al., 2011). Briefly, rats were trained to respond to a light stimulus in the stimulus hole in daily sessions that lasted 30 minutes or until 100 reinforcers were earned, whichever came first. To start, 1 pellet was delivered to the food magazine, and collecting the pellet began the first trial. This was followed by an inter-trial interval (ITI) of 5 seconds, after which the stimulus hole was illuminated for 5 seconds (the limited hold period [LH]). Nose-poke responses in the stimulus hole within the LH were rewarded by delivery of a pellet (“correct response”). Failure to respond within the LH period (“omission”) or responses made during the ITI (“premature response”) triggered a 5-second time-out, during which the house light was extinguished and no responses were rewarded.

Rats were food restricted to 80%–85% of free-feeding weight throughout the training and maintenance stages of the task. Rats were habituated to the chamber in one 30-minute session, during which sugar pellets were placed in the feeder and stimulus hole to elicit nose-pokes in these apertures. Training began the day after habituation and initially consisted of sessions with relatively long LH (60 seconds) and short ITI (1 second) to facilitate conditioning. Subsequent stages of training were conducted with increasingly shorter LH and longer ITI times until the animal reached a criterion of at least 80% correct responses with <20% omissions at ITI = 5 seconds, LH = 5 seconds, and time out = 5 seconds, maintained over 5 days. This typically required 5–6 weeks of training. During the test phases, rats were either exposed to the same conditions as the last stage of training (maintenance conditions with ITI = 5 seconds) or given a challenge test where a longer ITI was introduced (ITI = 8 seconds) that increases premature responses.

#### Dependent Measures

The measure of motor impulsivity was premature responses as a percent of total trials (premature responses/(correct responses + premature responses + omissions) × 100). Percent omissions (omissions/(correct + premature + omissions) × 100) was used as a measure of motivation to perform on the task. The number of reinforcers earned provided a measure of task competency. Although all rats were trained to a performance criterion of <20% omissions, during subsequent testing some rats may have scored higher than 20% omissions, especially after stress. Thus, the criterion for continued inclusion was adjusted to 35% omissions during testing. With these criteria, the following were excluded: 4 rats in the IS/ITI 5-second group, 1 rat in the chronic intermittent cold (CIC)/ITI 5-second group, and 1 rat in the CUS/ITI 5-second group.

### Acute Inescapable Stress (IS)

IS was carried out in shuttle boxes (Panlab, Barcelona, Spain) consisting of 2 equally sized chambers (25 cm × 25 cm × 28 cm) with independent grid floors connected by an automated guillotine door (8 cm × 10 cm) inside a sound-attenuating, dimly illuminated cabinet. The boxes were controlled by SHUTAVOID software. After a 5-minute habituation period during which the animal freely moved between compartments, the door was closed, and the animal was confined to 1 side where 60 footshocks (0.8 mA, 15 seconds) were delivered. The average ITI between shocks was 45 seconds (range = 30–60 seconds). IS was delivered on 2 consecutive days separated by 24 hours. Control rats were placed inside the shuttle boxes as above without receiving any footshock. Food restriction was maintained during the 2 days of acute IS.

An active avoidance test was used to validate the effectiveness of the IS (supplementary Methods). The test, repeated consecutively for 3 weeks, revealed a sustained IS-induced failure to escape the footshock (F_(1,16)_ = 26.9, *P* < .0001; supplementary Fig. 1).

### Chronic Intermittent Cold Stress

CIC stress was performed as previously described (Girotti et al., 2011). Rats were assigned to CIC stress or control groups. Cold-stressed animals were transported in their home cage with food, water, and bedding to a 4°C cold room for 6 h/d for 14 consecutive days. Control animals remained undisturbed in their home cage for the same period of time. Food restriction was suspended during the first 9 days of stress for both CIC and non-stress groups and resumed in the last 5 days of stress prior to 1-CSRTT testing.

### Chronic Unpredictable Stress (CUS)

CUS was conducted as previously described (Jett et al., 2015). A different acute stressor was administered daily, at various times of day, for 2 weeks. Following each session, rats recovered for 1 hour in an isolated room and then were transferred to clean cages and returned to housing. Unstressed controls were handled 1 min/d. Food restriction was suspended during the first 9 days of CUS and resumed in the last 5 days of stress prior to 1-CSRTT testing.

### Immunohistochemistry

One hour after the end of a test session, rats were anesthetized and perfused transcardially with 4% paraformaldehyde. Brains were cryoprotected in 30% sucrose and stored at −80°C until sectioning. Forty-micron-thick coronal sections were collected through the OFC (+4.70 to +2.70 from Bregma (Paxinos and Watson, 1997)) onto Superfrost Plus slides. Dried sections were rehydrated in phosphate-buffered saline and blocked for 1 hour in phosphate-buffered saline containing 0.3% Triton X-100, 5% normal donkey serum, and 1% bovine serum albumin. Sections were incubated with a rabbit anti–cFos antibody (1:1000 Synaptic Systems, Göttingen, Germany Cat# 226-003) at 4°C overnight, followed by a donkey anti-rabbit AlexaFluor488 conjugated secondary antibody (1:500, Jackson ImmunoResearch, West Grove, PA, USA Cat# 711-545-152). Slides were cover slipped with Prolong Gold Antifade mounting medium (ThermoFisher, *Waltham, MA USA* Cat# P36930). Fluorescent images were acquired using an Olympus IX81 (Tokyo, Japan) inverted confocal microscope and a 20× objective lens with FV10-ASW software (Olympus) and visualized with Fiji (National Institutes of Health, Bethesda, MD, USA). To quantify the number of Fos-positive cells, images of standard OFC fields (medial OFC, ventral OFC, lateral OFC, and agranular insular cortex) were collected bilaterally at anterior-posterior coordinates from +4.70 to +2.70 under the same conditions of illumination for all treatment groups. For each OFC subfield, the number of Fos-positive cells were counted and averaged along the AP extent. Data were collected from 4 rats per group.

#### Experiment 1. Fos Expression Induced in Orbitofrontal Cortex by Performance on 1-CSRTT

A total of 12 rats, assigned to no behavior, ITI 5, and ITI 8 groups (n = 4 per group) were trained in the 1-CSRTT until they reached stable performance (Fig. 1B). One group of 8 rats then continued with the same conditions (maintenance, ITI 5) for 2 more days, while another group was tested with the challenge condition (ITI 8). On the third day, one-half of the maintenance group were not tested (no behavior) and the other one-half was again tested at ITI 5. The challenge group was tested again at ITI 8. Testing at ITI 8 significantly increased premature responses compared with the maintenance group (*P* = .005; Fig. 1C), while omissions did not significantly differ (*P* = .124; Fig. 1D). One hour after the end of testing, rats were perfused and brains were processed for Fos immunohistochemistry.

#### Experiments 2–4. Effects of Stress on Motor Impulsivity

**Figure 2.**
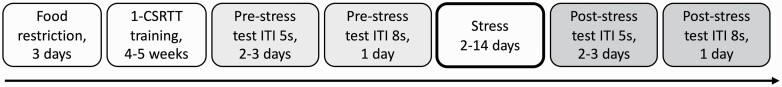
Diagrammatic representation of the experimental design of experiments 2–4.

The timeline for these experiments is depicted in Figure 2. A total of 17 rats were assigned to the IS experiment, 7 rats were assigned to the CIC experiment, and 8 rats to the CUS experiment. Following training, baseline (pre-stress) measures were taken for all rats at ITI 5 for 2–3 days followed by 1 day of challenge. Then rats underwent 2 days of IS, 14 days of CIC stress, or 14 days of CUS before retesting at ITI 5 for 2–3 days and ITI 8 for 1 day. Measures before and after stress were analyzed in a within-subject design.

### Statistical Analyses

Fos-positive cell counts were analyzed by 1-way ANOVA. When effects were significant, a Holm-Sidak post-hoc test was used for multiple comparisons. Behavioral measures on the active avoidance test and the 1-CSRTT were analyzed by repeated-measures ANOVA followed by Holm-Sidak post-hoc tests. The 1-CSRTT challenge test data were analyzed by paired, 2-tailed Student’s *t* test. All statistical analyses were performed in GraphPad Prism (Version 9) with significance determined at α < .05.

## Results

### Performance on 1-CSRTT Increases Fos Expression in OFC

We first determined whether performance in the 1-CSRTT under either maintenance conditions (using the same conditions of wait time the animal had mastered at the end of training, i.e., ITI 5 seconds) or challenge conditions (introducing a new, longer ITI that increases the likelihood of premature responses, i.e., ITI 8 seconds) would affect the activation of orbitofrontal cortex neurons as measured by Fos immunohistochemistry. Performance on the 1-CSRTT increased Fos expression in the OFC, but the magnitude of increase varied across OFC subregions. Fos expression was slightly and nonsignificantly increased in the medial OFC (MO, AP coordinates +4.20 to +3.70; Fig. 1I) after completion of the task at ITI 5 or ITI 8 (F_(2, 8)_ = 1.972, *P* = 0.2013; Fig. 1E,J). In the ventral OFC (VO, AP +3.70 to 2.70), Fos expression increased significantly at ITI 8 but not at ITI 5 (F_(2, 8)_ = 7.518, *P* = .0145; Fig. 1F,J). Finally, Fos expression increased significantly at both ITI 5 and ITI 8 in the lateral OFC (LO, AP +4.20 to +2.70, F_(2, 9)_ = 59.08, *P* < .0001; Fig. 1G,J) and agranular insula (AI, AP +3.70 to +2.70, F_(2, 9)_ = 19.48, *P* = .0005; Fig. 1H,J). Analyzed along the anterior-posterior axis, no significant effects were found in the MO and trending, but non-significant effects were found in the VO. Significant increases in Fos expression were found in the more posterior areas of LO and AI (+3.20 and +2.70) (supplementary Table 1). To eliminate the possibility that Fos counts were increased in the challenge condition simply because of increased time to complete the task (under well-trained conditions, rats may finish the ITI 5 task within 15–20 minutes, whereas they typically require the full 30 minutes for the challenge test), we analyzed the correlation between Fos counts and the time to finish in the ITI 5-second test, which has larger time variability, as some rats require 30 minutes and others perform in one-half the time. There was no correlation between time to finish the test and corresponding Fos counts in the MO (R^2^ = 0.35, *P* = .40), VO (R^2^ = 0.07, *P* = .74), LO (R^2^ = 0.41, *P* = .36), or AI (R^2^ = 0.002, *P* = .95).

### Acute IS Transiently Increases Motor Impulsivity in Maintenance Test

**Figure 3.**
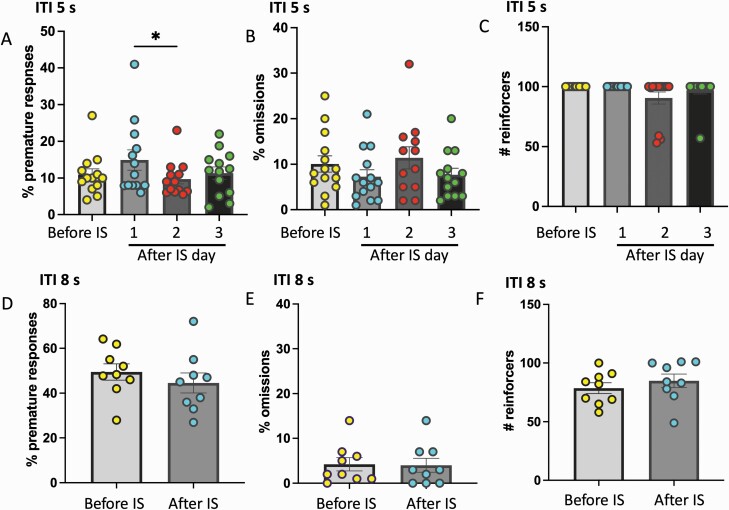
Acute inescapable shock (IS) stress increases premature responding at intertrial interval (ITI) 5 seconds (mastered task condition) but not at ITI 8 seconds (challenge condition). Percent of (A) premature responses, (B) omissions, and (C) number of reinforces in rats performing at ITI 5 seconds. Datapoints represent measures from individual rats before inescapable shock (IS; average of tests performed on 3 days before IS) and at 1, 2, and 3 days after IS. (D–F) Percent of premature responses, omissions, and number of reinforces in a subgroup of rats (n = 9) performing at ITI 8 seconds before and after IS. *P = .03, n = 13 per group.

Next, we explored the effects of stress on motor impulsivity. We began by examining the effects of a severe acute IS on performance in maintenance and challenge testing conditions. Following training, impulsivity measures were taken for all rats at ITI 5 for 3 days, and in a subgroup of animals this was followed by 1 day of challenge. Then all rats underwent 2 days of IS and were retested at ITI 5 and at ITI 8. ANOVA for the ITI 5 results indicated a significant overall effect (F_(3, 36)_ = 3.157, *P* = .0364; Fig. 3A). However, post-hoc tests revealed no significant differences comparing days after stress with pre-stress baseline, only between days 1 and 2 after stress. Thus, the effect of inescapable shock on premature responses in the ITI 5 test, if any, was modest at best. There were no significant differences in omissions (F_(3, 36)_ = 1.186, *P* = .3286) or in the number of reinforcers earned (F_(3, 36)_ = 1.265, *P* = .3008; Fig. 3B–C). Further, there were no significant effects of IS on premature responses (t_8_ = 0.8369, *P* = .427), omissions (t_8_ = 0.2229, *P* = .829), or reinforcers (t_8_ = 0.8354, *P* = .428) on the challenge test (Fig. 3D–F).

### CIC Stress has Opposing Effects on Impulsivity in the Maintenance and Challenge Tests

**Figure 4.**
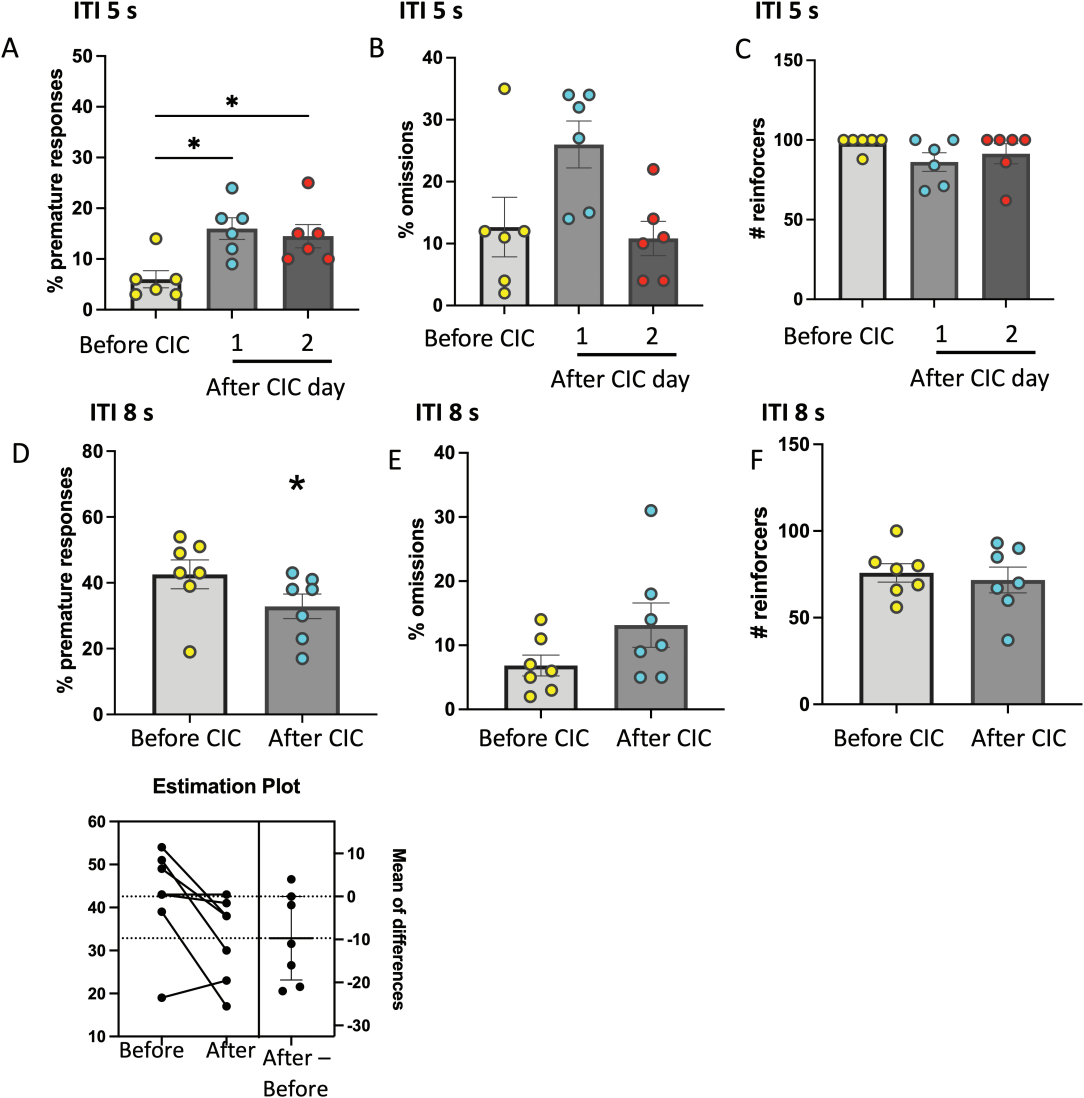
Chronic intermittent cold (CIC) stress produces a sustained increase in premature responding at intertrial interval (ITI) 5 seconds and reduces premature responding at ITI 8 seconds. Percent of (A) premature responses, (B) omissions, and (C) number of reinforces in rats performing at ITI 5 seconds. Datapoints represent measures from individual rats before CIC stress (average of tests on 2 days before stress) and at 1 and 2 days after CIC. (D–F) Percent of premature responses, omissions, and number of reinforces in rats performing at ITI 8 seconds before and after CIC stress. *P < .05, n = 67 per group.

Because neuronal activity increased in several OFC areas during performance in the 1-CSRTT, we tested whether CIC stress, which impairs OFC function (Lapiz-Bluhm et al., 2009; Adler et al., 2020), affected motor impulsivity in our test. CIC stress increased premature responding in maintenance conditions for at least 2 days after the end of stress (F_(2,10)_ = 7.920, *P* = .0087; Fig. 4A). ANOVA revealed a significant effect on omissions (F_(2,10)_ = 6.664, *P* = .027; Fig. 4B), although post-hoc analysis failed to identify specific significant comparisons. There were no effects on reinforcers obtained (F_(2,10)_ = 2.708, *P* = 0.1148; Fig. 4C). Premature responses in the challenge test decreased following CIC stress (t_6_ = 2.488, *P* = .025; Fig. 4D), with no effect on omissions (t_6_ = 2.013, *P* = .091; Fig. 4E) or reinforcers (t_6_ = 0.499, *P* = .635; Fig. 4F).

### CUS Increases Motor Impulsivity in Maintenance and Challenge Tests

**Figure 5.**
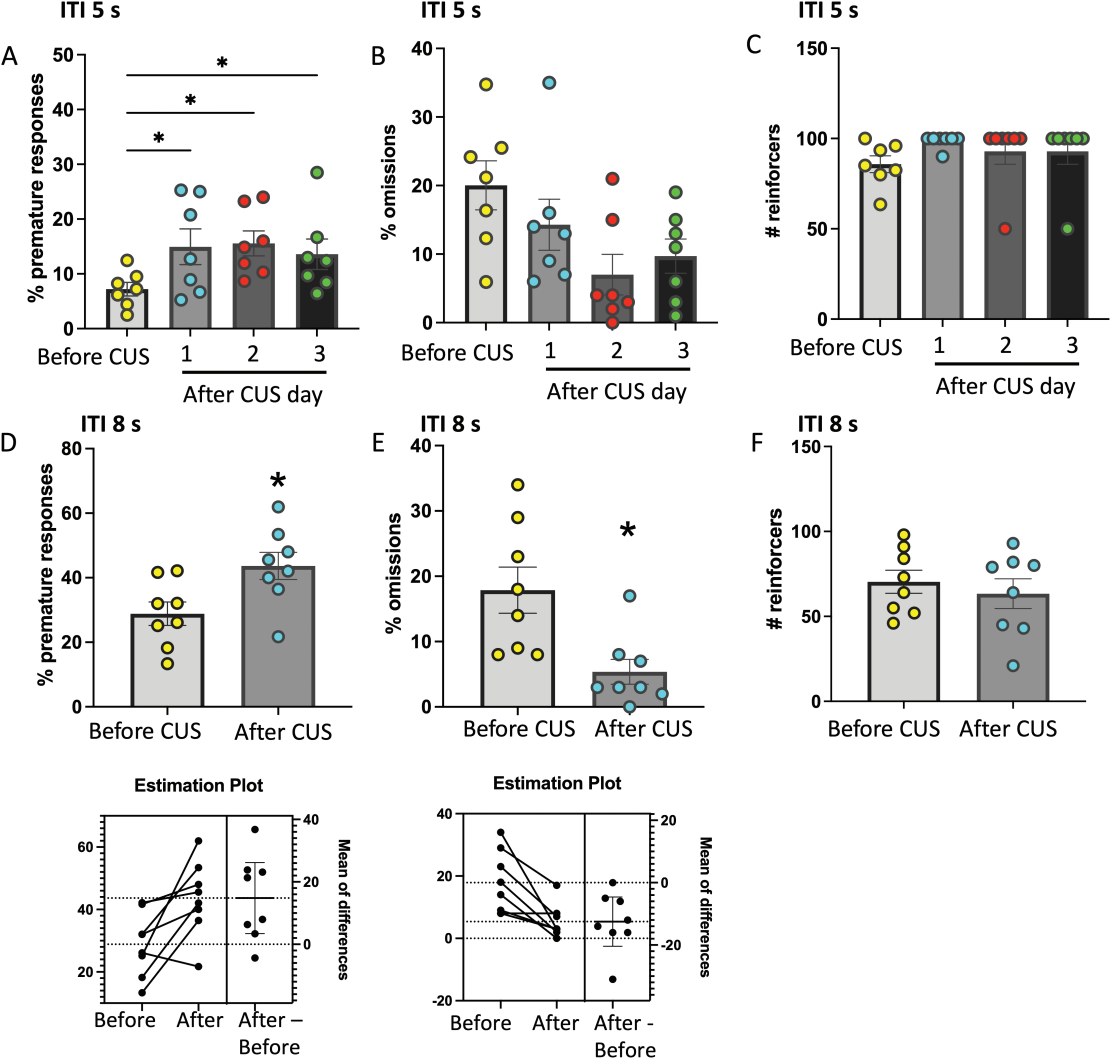
Chronic unpredictable stress (CUS) produces a sustained increase in premature responding at intertrial interval (ITI) 5 seconds and increases premature responding at ITI 8 seconds. Percent of (A) premature responses, (B) omissions, and (C) number of reinforces in rats performing at ITI 5 seconds. Datapoints represent measures from individual rats before CUS (average of tests on 3 days before stress) and at 1, 2, and 3 days after CUS. (D–F) Percent of premature responses, omissions, and number of reinforces in rats performing at ITI 8 seconds. *P < .05, n = 7–8 per group.

Finally, we examined the effects of CUS, a heterotypic psychogenic stress, on motor impulsivity. In the maintenance test, CUS increased premature responses, and this effect lasted for at least 3 days after the end of stress (F_(3,21)_ = 5.207, *P* = .0076; Fig. 5A). There was a significant effect of CUS on omissions (F_(3,21)_ = 4.619, *P* = .032; Fig. 5B), but no specific post-hoc comparisons were significant. There was no significant effect of CUS on reinforcers (F_(3,21)_ = 2.779, *P* = .125; Fig. 5C). In the challenge test, CUS increased premature responses (t_7_ = 3.081, *P* = .018; Fig. 5D) and decreased omissions (t_7_ = 3.745, *P* = .007; Fig. 5E) but did not affect reinforcers earned (t_7_ = 0.924, *P* = .386; Fig. 5F).

## Discussion

Using a modified version of the 1-CSRTT to study motor impulsivity, we showed that (1) OFC subregions are differentially activated during motor impulsivity tasks with different levels of inhibitory demand, and (2) different types of stress (severe acute uncontrollable, chronic metabolic, and chronic psychogenic stress) exerted different effects on motor impulsivity.

### Areas of OFC Involved in Motor Impulsivity

It is becoming increasingly evident that different subregions of the OFC have distinct roles in flexibility, decision-making, and impulse control and provide distinct contributions to pathophysiology (Mar et al., 2011; Fettes et al., 2017; Izquierdo, 2017; Hervig et al., 2020). However, much is still unknown about specific OFC areas involved in motor impulsivity. In this study, using a modified version of the 1-CSRTT, we explored activation of OFC subregions during tasks with different motor inhibitory demands: (1) a well-learned task (maintenance conditions, ITI 5s) and (2) a novel challenge task requiring a new and longer wait time (ITI 8s). Overall, performance in the 1-CSRTT increased Fos expression in lateral OFC and agranular insular cortex, both in the well-learned and challenge tasks. In ventral OFC, significant increases were observed only during challenge. Finally, medial OFC Fos expression was more modestly and not significantly changed by performance in the 1-CSRTT. Thus, with respect to motor impulsivity in the 1-CSRTT, there appears to be a “gradient” of increasing neuronal activation along the OFC medio-lateral axis. The more lateral areas, LO and AI, that were activated during both the maintenance test and challenge tests may be involved in the basic performance of the task. Indeed, both LO and AI and their major projection targets in the central and lateral segments of dorsal striatum and basolateral amygdala, respectively (McDonald, 1998; Hoover and Vertes, 2011; Heilbronner et al., 2016), have well-documented roles in response inhibition and reward processing. LO and AI are instrumental in reversal learning (Schoenbaum et al., 1999, 2000, 2003), delayed discounting (Zeeb et al., 2010; Mar et al., 2011), and other decision-making processes involving cue-based outcome predictions and willingness to wait for reward (Lak et al., 2014). This is therefore consistent with the increased activation of these areas observed in this task of motor inhibition.

In contrast to LO and AI, VO was recruited more during the challenge test, suggesting a possible role of this region in flexibly adapting the wait response to a change in task requirements. Indeed, other evidence suggests that VO is required for response inhibition but specifically in value learning when outcomes are not easily predicted or in situations of delay uncertainty (Meyer and Bucci, 2016; Hardung et al., 2017; Stolyarova and Izquierdo, 2017). Less activation in the MO is consistent with a more substantial role of this subregion and its target areas (ventral striatum and nucleus accumbens) in choice impulsivity (delay and probabilistic discounting) and with the fact that MO is required for maintaining a memory of reward value to guide actions in situations of uncertain outcomes (Bradfield et al., 2015; Dalton et al., 2016; Wang et al., 2019; Jenni et al., 2022), conditions not explicitly tested in our experiments. Integrating anatomical and functional observations, it has been suggested that more medial subregions of rat OFC provide affective and motivational contributions to decision-making, whereas lateral subregions support sensory integration (i.e., associations of stimuli/cues and sensory events to outcomes) (Izquierdo, 2017). In this view, therefore, it would make sense that a relatively simple and automatic test of motor impulsivity may not elicit a robust recruitment of affective circuits but may rely more heavily on sensory information to predict reward outcomes. Besides medial-lateral functional parcellation, OFC is also differentiated along its anterior–posterior axis (Barreiros et al., 2021). We found that within LO and AI, significant increases in Fos expression at ITI 5s and 8s were observed primarily in the posterior sections (AP +3.2 to +2.7). Posterior areas of LO and AI have been shown to harbor the majority of reciprocal connections to basolateral amygdala (BLA) ; these areas also receive substantive projections from MO and medial prefrontal cortex (mPFC) in contrast to anterior LO, where corticocortical innervation is sparse (Hoover and Vertes, 2011; Barreiros et al., 2021).

Taken together, these anatomical findings provide correlational evidence that activation of specific OFC areas is associated with behavioral inhibition, and this can inform future studies aimed at determining the involvement of specific OFC circuits in motor inhibition. Although we have focused on OFC in this study, it is important to acknowledge that other brain regions also play an important role in the control of motor impulsivity. For example, basolateral amygdala, which has bidirectional connections with OFC, dorsal and ventral striatum, which are important OFC efferent targets, ventromedial prefrontal cortex, and subthalamic nucleus have all been implicated in the control of motor impulsivity (Muir et al., 1996; Dalley et al., 2002; Eagle and Baunez, 2010; Donnelly et al., 2015; Jenni et al., 2022). Recently, a claustrum-mPFC pathway has been shown to regulate impulsive responding in the 5-CSRTT (Liu et al., 2019), Thus, future experiments targeting subpopulations of neurons in the OFC and/or projection-specific input and output pathways, as well as other non-OFC areas, will provide a more refined understanding of the circuits and systems underlying the regulation of motor impulsivity.

Finally, global lesion studies suggest the OFC is necessary for effective response inhibition. However, more selective inactivation of the medial and lateral OFC revealed possible opposing functions (Mar et al., 2011). Thus, it is possible that neuronal activity in some subregions of the OFC may enable the expression of behavior (in this case, impulsive motor responding), while neuronal activity in other subregions may in fact suppress the same behavior (motor inhibition). Further work is necessary to discriminate between these alternatives.

### Effects of Stress on Motor Impulsivity

The impact of stress on premature responding differed depending on the type of stress and the task (maintenance or challenge). Acute IS had a modest effect, if any, on the well-learned maintenance task and did not affect premature responding on the challenge task. One consideration is that challenge was presented 4 days after the end of stress, and this may have exceeded the duration of effect of this acute stressor. It is possible that IS may have had an impact on the challenge task if it had been tested 24 hours after stress. However, we are inclined to dismiss this possibility for the following reasons. IS has been used to model intense and unavoidable stress that leads to learned helplessness, a behavioral construct wherein the individual perceives itself unable to cope with new challenges (Maier and Seligman, 1976). Accordingly, escape deficits in the active avoidance test are used as a measure of learned helplessness following inescapable shock (Maier and Seligman, 1976). In our study, rats showed sustained (up to 3 weeks) increases in escape failures in the active avoidance test (supplementary Fig. 1). Together, this suggests that whereas this stressor was effective in producing lasting behavioral deficits associated with memory of trauma-related cues (contextual fear memory), its impact on motor impulsivity was very modest, possibly because performance in this test does not rely on circuits influenced by affective status (medial OFC). It has been shown that IS-induced escape deficits are sustained for weeks only when the shock was delivered in the same apparatus used for the avoidance test (Maier and Watkins, 2005; Hunziker and Dos Santos, 2007; Zazpe et al., 2007). If the shock was administered in a context other than that used to perform the active avoidance test, IS-induced deficits in escape response were short lived, lasting up to 48 hours (Maier, 2001).

By contrast with acute IS, chronic stress caused more robust and prolonged increases in premature responding. CIC stress and CUS both increased premature responding on the maintenance task, and these effects were sustained for at least 48–72 hours after stress, suggesting that both stressors impacted circuits that control motor inhibition. However, interesting differences were observed in the challenge test: rats exposed to CUS continued to show impaired motor inhibition, whereas CIC-stressed rats actually showed improved motor control. We have previously described effects of CIC stress in another test of goal-directed executive function: reversal learning with olfactory and tactile cues. Reversal learning requires the ability to suppress a previously rewarded but now nonrewarded response and to update behavioral strategies to the new contingency. Because it engages behavioral inhibition, reversal learning models constructs relevant to understanding impulsive and compulsive behavior (Izquierdo and Jentsch, 2012). Importantly, reversal learning, like the inhibition of impulsive responding, relies on OFC integrity (McAlonan and Brown, 2003). We have shown that CIC stress produces selective deficits in reversal learning (Lapiz-Bluhm et al., 2009; Patton et al., 2017; Adler et al., 2020). Functionally, CIC stress increased OFC responses to afferent stimulation (Adler et al., 2017), whereas inducing long-term depression in the lateral OFC rescued CIC-induced reversal learning deficits (Adler et al., 2020). These results indicate that CIC stress may produce a state of hyperactivity and increased responsivity in the OFC that negatively impacts reversal learning. In light of these data, we hypothesize that CIC stress may disrupt maintenance of the well-learned task and increase motor impulsivity because it induces hyperactivity in regions like the MO and VO that are not normally activated during the maintenance task. Conversely, a state of increased activity in these regions of the OFC may be conducive to better performance when the task requirements change or become uncertain (challenge). The latter situation is perhaps similar to studies that addressed LO involvement in outcome prediction during gambling tasks. Inactivation of LO decreased the propensity of rats to wait for reward, and this was not due to inability to estimate elapsed time. Interestingly, this phenomenon was observable only in a situation of “wagering” and not during well-learned tasks with predictable outcomes (Lak et al., 2014). Thus, speculatively, increased activity in LO after CIC stress may produce a faster adaptation to increased waiting requirements in the challenge test. Future mechanistic intervention studies are needed to test this hypothesis. It is also possible that other areas involved in control of impulsive responding, such as the ventral striatum or basolateral amygdala, that are also stress responsive (Ostrander et al., 2009; Spring et al., 2021) may contribute to the observed effects of stress.

Intuitively, performance in the challenge task might be likened to flexible responding because the rat has to let go of a previously learned timing cue to adapt to a new one, yet we did not see the same CIC-induced deficits we observed in reversal learning. However, we should also consider some fundamental differences between the 2 tests: the primary goal of 1-CSRTT is to test how automatic or habitual responses are inhibited within a context of reward outcomes that quickly become predictable, and in this sense the decision-making process may be less complex (and therefore less susceptible to disruption by CIC stress) than the reward/outcome evaluation process required for reversal learning.

Unlike CIC stress, CUS also impaired motor inhibition in the challenge condition, measured 4 days after the end of stress. CUS, like CIC stress, produces deficits in reversal learning. However, CUS also impairs extradimensional set shifting, a form of cognitive flexibility dependent on the medial prefrontal cortex (Birrell and Brown, 2000; Bondi et al., 2010; Jett et al., 2015). Medial prefrontal and cingulate cortex have well-documented roles in impulsive responding (Risterucci et al., 2003; Gourley and Taylor, 2016). In particular, the ventral subregions (infralimbic cortex) are involved in motor impulsivity (Chudasama et al., 2003). Recently, a role for a claustrum-mPFC pathway in regulating premature responding has been reported (Liu et al., 2019). Thus, it is possible that CUS produces more substantial increases in premature responding in both well-learned and novel wait conditions because of its ability to disrupt multiple cortical areas involved in impulse control. Future work will address how chronic stress may alter activity in the specific circuits involving the OFC and mPFC that regulate motor impulsivity.

## Conclusions

Different subregions of the OFC were activated during a task requiring inhibition of motor impulsivity performed under well-learned or novel timing conditions. Moreover, acute IS produced a modest effect on premature responding, whereas 2 chronic stressors, CIC stress and CUS, increased premature responding in ways that may depend on specific circuits and brain areas affected by each stressor. Future work will focus on the characterization of specific neuronal substrates of motor impulse control affected by stress to increase understanding of the mechanisms underlying the impact of stress on the pathophysiology of impulsivity.

## Supplementary Material

pyac062_suppl_Supplementary_Material

## Notes

This work was supported by the National Institute of Mental Health (research grant R01MH053851) and by the US Department of Veterans Affairs Biomedical Laboratory Research and Development Program (merit award I01BX000559). The contents of this paper do not represent the views of the Department of Veterans Affairs or the US Government.

## Statement of Interest

The authors have no financial or nonfinancial disclosures to report directly related to this work. Within the past 3 years, Dr Morilak has received in-kind support from Lundbeck that is not related to the work reported in this paper.
