# Supplementary material for: Role of Orbitofrontal Cortex and Differential Effects of Acute and Chronic Stress on Motor Impulsivity Measured With 1-Choice Serial Reaction Time Test in Male Rats
Source: Int J Neuropsychopharmacol. 2022 Sep 10;25(12):1026–36. doi: 10.1093/ijnp/pyac062 (PMC9743967; doi:10.1093/ijnp/pyac062)
Supplement: pyac062_suppl_Supplementary_Material [file pyac062_suppl_supplementary_material.docx]

**Supplementary Material**

**Methods**

**Active avoidance test**

Rats (N=18) were exposed to a 5-min habituation period in the same chamber where the IS was applied. This was followed by 30 escape trials during which the gate separating the two halves of the shuttle box opened for 5 s prior to shock onset, followed by 0.65 mA footshock for a maximum 15 s. The average ITI was 60 s (range, 20–100 s). The first 5 escape trials required one crossing for termination of the foot shock (fixed ratio 1; FR-1). For the remaining 25 trials, two crossings were required for shock termination (FR-2). The dependent measure was the number of escape failures, i.e., failure to cross to the other compartment to terminate shock, in both the FR-1 and FR-2 trials.

**Figure legend**

Supplementary Fig. S1. **Two sessions of inescapable foot shock caused sustained deficits on escape performance in the active avoidance test.** Data represent individual values as well as group mean number of escape failures ±SEM. ***p= 0.0072, ****p< 0.0001, Holm-Sidak’s post-hoc test, n= 6-12 per group.


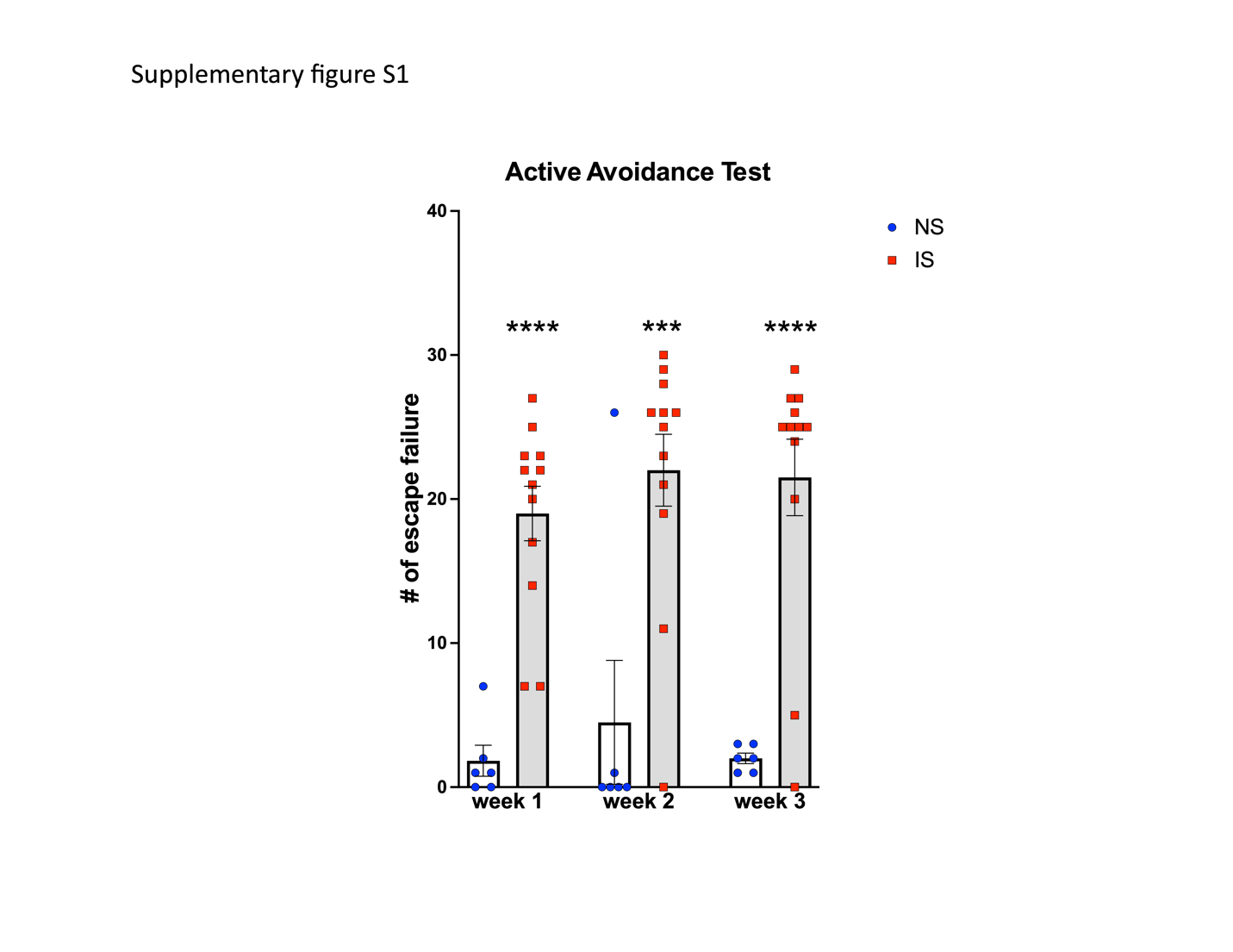


| **Table S1. One-way ANOVA of Fos counts in OFC subregions by anterior-posterior coordinate. Significant p values are in bold font.** | | | | |
| --- | --- | --- | --- | --- |
|  | + 4.20 | + 3.70 | +3.20 | +2.70 |
| MO | F (2, 5) = 0.133, P=0.878 | F (2, 7) = 1.474, P=0.297 |  |  |
| VO | F (2, 5) = 1.142, P=0.390 | F (2, 7) = 2.738, P=0.132 | F (2, 8) = 4.146, P=0.058 | F (2, 3) = 2.214, P=0.257 |
| LO | F (2, 4) = 1.582, P=0.311 | F (2, 7) = 4.022, P=0.069 | F (2, 8) = 133.5, **P<0.0001** | F (2, 7) = 12.53, **P=0.005** |
| AI |  | F (2, 6) = 2.447, P=0.167 | F (2, 8) = 10.00, **P=0.007** | F (2, 8) = 32.50, **P=0.0006** |
